# Comparative Analysis of the Characteristics of Two Hardy Kiwifruit Cultivars (*Actinidia arguta* cv. Cheongsan and Daebo) Stored at Low Temperatures

**DOI:** 10.3390/plants13162201

**Published:** 2024-08-09

**Authors:** Hyun Ji Eo, Chul-Woo Kim, Uk Lee, Yonghyun Kim

**Affiliations:** Special Forest Resources Division, National Institute of Forest Science, Gwonseon-gu, Suwon 16631, Republic of Korea; eohyunji1030@korea.kr (H.J.E.); futuretree@korea.kr (C.-W.K.); rich26@korea.kr (U.L.)

**Keywords:** hardy kiwifruit, low temperature, storage, antioxidant, ascorbic acid

## Abstract

A cold storage system is useful for maintaining the quality of hardy kiwifruit. However, extended cold storage periods inevitably result in cold stress, leading to lower fruit marketability; the severity of chilling injury depends on fruit types and cultivars. In this study, the impact of cold storage conditions on the physicochemical properties and antioxidant capacity of two phenotypically different hardy kiwifruit cultivars—‘Cheongsan’ (large type) and ‘Daebo’ (small type)—stored at low (L; 3 °C, relative humidity [RH]; 85–90%) and moderate-low (ML; 5 °C, RH; 85–90%) temperatures was determined. Significant differences in fruit firmness and titratable acidity between treatments L and ML were observed in both cultivars during the experimental storage period. Meanwhile, the browning and pitting rates of the ‘Cheongsan’ fruits in treatment L increased for 8 weeks compared with those of the ‘Daebo’ fruits in treatments L and ML; nonetheless, fruit decay was observed in the ‘Daebo’ fruits in treatment ML after 6 weeks. The total chlorophyll, carotenoid, flavonoid, and ascorbic acid concentrations as well as the antioxidant activities of both the cultivars significantly differed between treatments L and ML. After 2 weeks of storage, the ‘Cheongsan’ fruits in treatment L had lower antioxidant activities and ascorbic acid content than those in treatment ML. These results demonstrate that the quality attributes and antioxidant activity of hardy kiwifruit are influenced by the low-temperature storage conditions and the specific kiwifruit cultivars. Our findings suggest that optimal cold storage conditions, specific to each hardy kiwifruit cultivar, promise to maintain fruit quality, including their health-promoting compounds, during long-term storage.

## 1. Introduction

The hardy kiwifruit (*Actinidia arguta*; Family Actinidiaceae) has a smooth, edible skin and is smaller than fuzzy kiwifruit varieties such as ‘Hayward’ [1]. It is cultivated in Northeast Asia, Australia, New Zealand, and several European countries [2,3,4]. The hardy kiwifruit is rich in essential minerals, vitamin C, flavonoids, and phenolics, which contribute to its health-promoting properties. Due to its high nutritional value, the market demand for hardy kiwifruit has gradually yet consistently increased [5]. However, like other climacteric fruits, the softening of the peel and pulp of the hardy kiwifruit during ripening significantly reduces its shelf life [4,6]. Therefore, delaying the ripening process is crucial for maintaining fruit quality during an extended storage period.

A cold storage system is commonly used to preserve the quality and extend the shelf life of various crops and fruits. Hardy kiwifruits harvested at the firm and mature stage can be stored at 0 °C for up to 2 months [6]. However, the maintenance of fruit quality during extended storage periods is not guaranteed, as chilling injury may occur under suboptimal cold storage conditions. For instance, kiwifruits (*Actinidia deliciosa* cv. Hayward) are more susceptible to chilling injury at temperatures near the freezing point than at temperatures above 1 °C [7]. In an experiment, chilling injury symptoms such as browning and pitting were more pronounced in kiwifruits stored continuously at 0 ± 0.5 °C than in those subjected to low-temperature conditioning—a treatment designed to mitigate chilling injury prior to cold storage [8]. Thus, it is necessary to identify the optimal cold storage conditions for maintaining the quality of hardy kiwifruit during long-term storage.

Hardy kiwi cultivars, which vary in fruit size, are categorized into early-, mid-, and late-maturing species based on their harvest times [9]. Despite sharing genetic similarities, it is possible that hardy kiwi cultivars respond differently to cold environments due to their distinct morphological and biochemical traits. Hence, in this study, we hypothesized that different hardy kiwifruit cultivars, which vary significantly in their morphology, would exhibit distinct responses to cold storage, thereby impacting their physicochemical and metabolic properties over the storage period. Therefore, the differences in the physicochemical properties and antioxidant content of the ‘Cheongsan’ (small type) and ‘Daebo’ (large type) cultivars stored at low (L; 3 °C, relative humidity [RH]; 85–90%) and moderate-low (ML; 5 °C, RH; 85–90%) temperatures were determined. The findings of this study contribute to the development of strategies aimed at maintaining the fruit quality of specific hardy kiwi cultivars during long-term cold storage.

## 2. Results

### 2.1. Effect of Storage Temperatures on the Quality Attributes of ‘Cheongsan’ and ‘Daebo’ Hardy Kiwifruits

**Figure 1:**
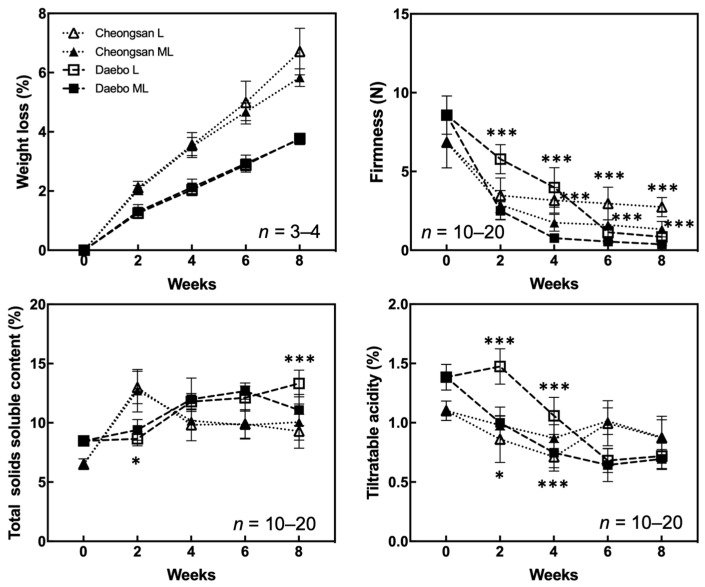
Changes in the physicochemical properties of the ‘Cheongsan’ and ‘Daebo’ hardy kiwi cultivars during cold storage. Weight loss, firmness, total soluble solids content, and titratable acidity were measured for 8 weeks. The asterisks indicate a significant difference for each cultivar between treatments L and ML according to Student’s *t*-test (* *p* < 0.05 and *** *p* < 0.001).

We investigated the quality attributes of the hardy kiwifruit cultivars ‘Cheongsan’ and ‘Daebo’ stored at L and ML to determine the effect of each cold storage condition on fruit quality changes. The ‘Cheongsan’ and ‘Daebo’ fruits gradually lost weight over an 8-week period; however, there was no significant difference in fruit weight loss between treatments L and ML (Figure 1). Nevertheless, the ‘Cheongsan’ fruits lost twice as much weight as that of the ‘Daebo’ fruits. ‘Daebo’ stored at L maintained significantly higher firmness for up to 8 weeks compared to those stored at ML despite the continuous decrease in firmness during the storage period. Otherwise, the firmness of the ‘Cheongsan’ fruits in treatments L and ML rapidly declined within 2 weeks; however, it remained relatively stable until the 8th week of the experiment, with a significant difference between treatment L and ML. The total soluble solids content (TSSC) of the ‘Cheongsan’ fruits did not significantly differ between treatments L and ML; it initially increased until the 2nd week then decreased and stabilized up to the 8th week. Meanwhile, the TSSC of the ‘Daebo’ fruits in both treatments gradually increased over 6 weeks; however, after 8 weeks, the TSSC of the ‘Daebo’ fruits in treatment ML was significantly lower than that of the fruits in treatment L.

**Figure 2:**
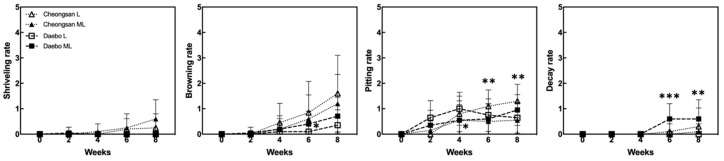
Incidence of physiological disorders in the hardy kiwifruits during cold storage. Shriveling, browning, pitting, and decay rates were evaluated based on a 6-point scale: 0 = 0%, 1 = 1–20%, 2 = 21–40%, 3 = 41–60%, 4 = 61–80%, and 5 = 81–100% of the corresponding affected area on the fruit surface. The asterisks indicate a significant difference for each cultivar between treatments L and ML according to Student’s *t*-test (* *p* < 0.05, ** *p* < 0.01, and *** *p* < 0.001; *n* = 17–20).

**Figure 3:**
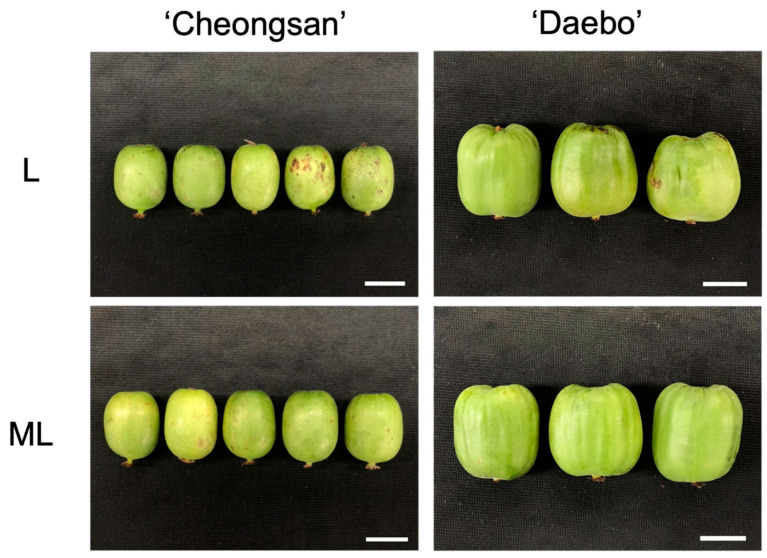
‘Cheongsan’ and ‘Daebo’ fruits stored at 3 °C and 5 °C after 6 weeks of storage. Bars indicate 2 cm.

The ‘Cheongsan’ fruits in treatment L had a significantly lower titratable acidity (TA) than those in treatment ML for the first 4 weeks, while the TA of the ‘Daebo’ fruits in L gradually decreased for up to 4 weeks. Physiological disorders were apparent in the two cultivars in treatments L and ML for 8 weeks (Figure 2). The degree of shriveling, browning, pitting, and decay in the ‘Cheongsan’ and ‘Daebo’ fruits gradually increased throughout the 8-week experimental period. Specifically, the browning rate of the ‘Daebo’ fruits in treatment L was significantly lower than that of the fruits in treatment ML after 6 weeks of storage. Meanwhile, the pitting rates of the ‘Cheongsan’ and ‘Daebo’ fruits in treatment L were significantly higher after 6 and 4 weeks, respectively (Figure 2 and Figure 3). The decay rate of the ‘Daebo’ fruits in treatment ML significantly increased after 6 weeks.

**Figure 4:**
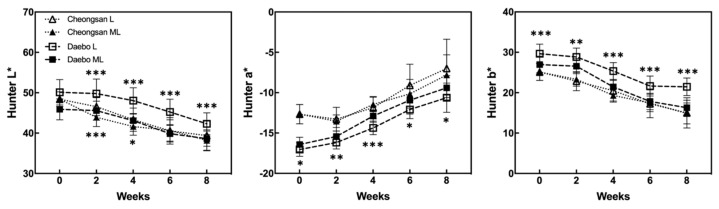
Changes in the Hunter values were monitored during the storage period and compared between the storage temperatures. The asterisks indicate a significant difference for each cultivar between treatments L and ML according to Student’s *t*-test (* *p* < 0.05, ** *p* < 0.01, and *** *p* < 0.001; *n* = 20).

The color characteristics of ‘Cheongsan’ and ‘Daebo’ were measured during storage (Figure 4). Although the Hunter L* (brightness) values gradually decreased in both the cultivars, the Hunter L* values of the ‘Cheongsan’ and ‘Daebo’ fruits in treatment L were higher over 4 and 8 weeks, respectively, than those of the cultivar fruits in treatment ML. The Hunter a* (green < red) values of both the ‘Cheongsan’ and ‘Daebo’ fruits increased after cold storage (Figure 4). There was no significant difference in the Hunter a* values between the ‘Cheongsan’ fruits in treatments L and ML; meanwhile, the ‘Daebo’ fruits in treatment L had significantly lower Hunter a* values than those in ML. The Hunter b* (blue < yellow) values of the ‘Cheongsan’ and ‘Daebo’ fruits decreased during cold storage. The Hunter b* values of the ‘Cheongsan’ fruits were not significantly different between treatments L and ML; however, the ‘Daebo’ fruits in L had significantly higher Hunter b* values than those in ML for 8 weeks. These results indicate that the postharvest ripening process of hardy kiwifruit is influenced by storage temperature; specifically, storage at a lower temperature can delay the ripening process, as evidenced by higher fruit firmness, TA, and color characteristics in treatment L than in ML.

### 2.2. Changes in Starch and Sugar during the Storage at Low Temperatures

**Figure 5:**
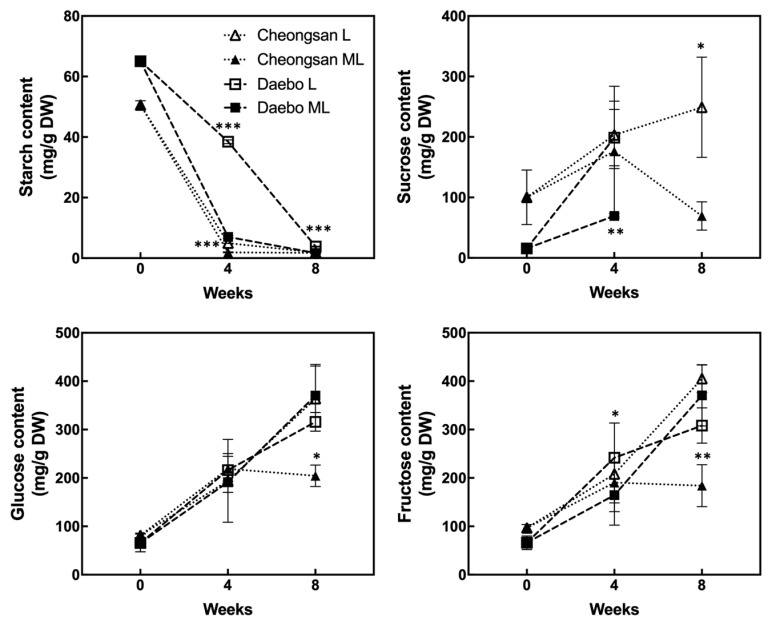
Changes in the starch and soluble sugar content in the hardy kiwifruits during storage. The asterisks indicate a significant difference for each cultivar between treatments L and ML according to Student’s *t*-test (* *p* < 0.05, ** *p* < 0.01, and *** *p* < 0.001; *n* = 3).

Starch degradation and soluble solids accumulation are the major ripening traits of kiwifruits. As shown in Figure 5, the starch content of the ‘Cheongsan’ fruits sharply decreased after 4 weeks of storage under both the L and ML treatments. However, the ‘Daebo’ fruits exhibited different levels between the L and ML treatments, with the starch content in treatment L being significantly higher than in treatment ML. These data indicate that starch degradation in the ‘Cheongsan’ fruits is relatively insensitive to variations in low temperature. The sucrose level in the ‘Cheongsan’ fruits gradually increased during the storage period under treatment L, whereas it did not increase under treatment ML after 4 weeks. In the ‘Daebo’ fruits, treatment L showed a higher level of sucrose compared to treatment ML; however, sucrose was not detected in either treatment after 8 weeks of storage. Interestingly, although the levels of glucose and fructose in the ‘Daebo’ fruits gradually increased in both treatments during the storage period, these levels did not increase in the ‘Cheongsan’ fruits under treatment ML after 8 weeks of storage. Based on these data, soluble sugar accumulation is affected by the storage temperature depending on the hardy kiwifruit cultivar.

### 2.3. Effect of Storage Temperatures on the Biochemical Properties of ‘Cheongsan’ and ‘Daebo’

**Figure 6:**
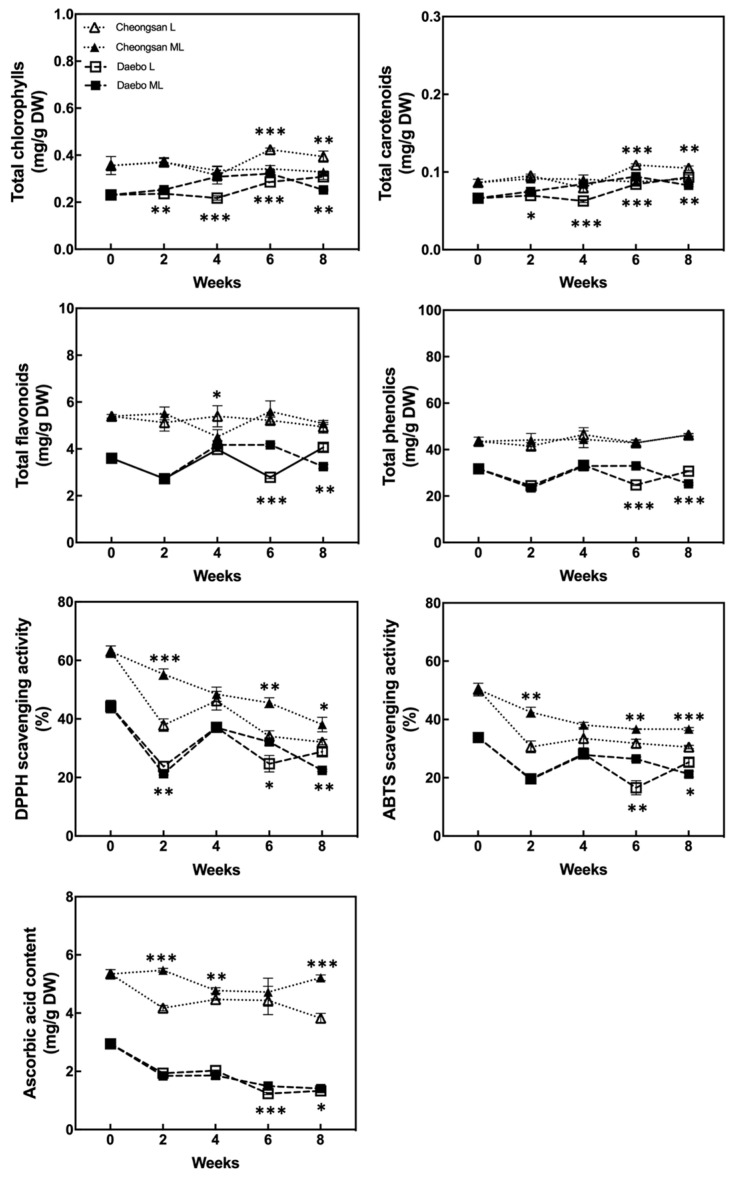
Changes in the content of the health-promoting compounds and antioxidant activity. The asterisks indicate a significant difference for each cultivar between treatments L and ML according to Student’s *t*-test (* *p* < 0.05, ** *p* < 0.01, and *** *p* < 0.001; *n* = 3).

The total chlorophyll, carotenoid, flavonoid, and phenolic concentrations of the hardy kiwifruits are shown in Figure 6. The total chlorophyll and carotenoid content of the ‘Cheongsan’ fruits in treatment L remained stable for 8 weeks, whereas that of the ‘Cheongsan’ fruits in treatment ML decreased after 6 weeks. The total chlorophyll and carotenoid content of the ‘Daebo’ fruits remained stable for 8 weeks; it was significantly higher after 4 weeks in treatment ML compared to treatment L, but after 8 weeks, treatment L exhibited higher content than treatment ML. The total flavonoid and phenol content of both the cultivars also remained stable during cold storage despite significant fluctuations at certain times. More antioxidant compounds were observed in the ‘Cheongsan’ fruits than in the ‘Daebo’ fruits during the storage period.

The antioxidant activity of the two cultivars was estimated using the 2, 2′-diphenyl-2-picrylhydrazyl (DPPH) and 2, 2′-azino-bis (3-ethylbenzothiazoline-6-sulfonic acid) (ABTS) methods [10,11]. The DPPH- and ABTS-scavenging activities of both cultivars gradually decreased over 8 weeks (Figure 6). The ‘Cheongsan’ fruits in treatment L had significantly lower antioxidant activity than those in ML after 2 weeks. Meanwhile, the ‘Daebo’ fruits in treatment L exhibited a significantly lower antioxidant activity after 6 weeks. The trend in the ascorbic acid content of the two cultivars was similar to that of the antioxidant activity (Figure 6). The ascorbic acid content of both the cultivars in treatment L was significantly lower than that in ML, in which the extent of the difference is larger in the ‘Cheongsan’ fruits. The ascorbic acid content of the ‘Cheongsan’ fruits remained stable in treatment ML. These results indicate that changes in the levels and activities of the antioxidants in hardy kiwifruit are influenced by the cultivar and storage temperatures, as the antioxidant activity and ascorbic acid content of ‘Cheongsan’ exhibited a considerable decrease in treatment L compared to ML during storage. In addition, it is possible that ascorbic acid played a major role in the antioxidant activity of the hardy kiwifruit compared with chlorophyll, flavonoids, and phenols.

## 3. Discussion

Chilling injury symptoms such as epidermis pitting, water-soaked and grainy tissue appearance, and lignification manifest during the cold storage of kiwifruit. The degree of these symptoms depends on the cultivar, stage of fruit maturity, and storage duration [12]. Specifically, genetic differences among fruit cultivars affect their response to cold stress by mediating metabolomic dynamics, antioxidant activities, and other physiological processes that contribute to fruit quality and storage life [13,14,15,16]. For example, *Actinidia chinensis* var. chinensis ‘Hongyang’ (a cold-sensitive cultivar) showed a higher chilling injury index with more lignified flesh tissue than ‘Xuxiang’ during cold storage at 0 °C [12]. The difference in cold injury between the two cultivars was dependent on the key enzyme activities involved in lignin synthesis. Additionally, variations in the metabolomic and transcriptomic characteristics in response to cold environments are involved in regulating the freezing tolerance of kiwifruit genotypes [15,17]. In this study, the differences in the responses of the ‘Cheongsan’ and ‘Daebo’ hardy kiwifruit cultivars to low-temperature storage were examined. The two fruit cultivars are phenotypically different based on their size (Figure 3); the fresh weight of ‘Daebo’ is approx. twice that of ‘Cheongsan’ [9]. We hypothesized that these two cultivars have distinguishable responsiveness to cold storage based on their genetic traits, which affect their physiological and metabolic properties during storage. Our results showed that the responses of the two cultivars to cold storage differed. Specifically, fruit weight loss increased dramatically in the ‘Cheongsan’ fruits compared with the ‘Daebo’ fruits, and differential changes in the other physicochemical properties were also observed during cold storage (Figure 1). Moreover, the incidence of physiological disorders, such as shriveling, browning, and pitting, was higher in the ‘Cheongsan’ fruits than in the ‘Daebo’ fruits regardless of the temperature (Figure 2). As the ‘Cheongsan’ and ‘Daebo’ fruits exhibited different characteristics during the cold storage period, it is reasonable to suggest that they have distinct genetic traits, including their size, in response to cold environments. Consequently, ‘Cheongsan’ appears to be a relatively cold-susceptible cultivar compared to ‘Daebo’. Although the specific genetic characteristics involved in the cold storage responses of ‘Cheongsan’ and ‘Daebo’ need further elucidation, it can be speculated that their genetic traits are associated with their different responses to cold storage conditions.

Moisture loss during storage is a crucial factor affecting the quality of postharvest products. Moisture loss is a complex process involving the physiological, biochemical, and physicochemical properties of postharvest products; it is also affected by postharvest treatment and handling [18]. Moisture loss occurs not only through surface microcracks and openings but also across the intact surface layer of the cuticle [19]. Therefore, the extent of moisture loss during cold storage can be influenced by fruit types and cultivars. Moreover, environmental conditions such as temperature and humidity affect the rate of water loss and physiological changes in fruits [14]. Low-temperature storage upregulates stress-responsive genes, blocks the signal transduction of ethylene-related processes, and affects both primary and secondary metabolism [20,21]. As low-temperature conditions delay metabolic processes and maintain membrane stability, fruit weight loss is relatively reduced during low-temperature storage. As the weight loss of the ‘Cheongsan’ fruits is twice as much as that of the ‘Daebo’ fruits in both treatment L and ML (Figure 1), primary and secondary metabolic processes may be more active in ‘Cheongsan’ than in ‘Daebo.’ This notion is partly supported by the rapid starch degradation observed in ‘Cheongsan’ fruits during 4 weeks of storage under both treatment L and ML (Figure 5). Additionally, TSSC dramatically increased in ‘Cheongsan’ after 2 weeks of storage compared to the ‘Daebo’ fruits despite the level of soluble sugars comparable between the two hardy kiwifruits after 4 weeks of storage under treatment L (Figure 5 and Figure 6). On the other hand, it is possible that ‘Cheongsan’ and ‘Daebo’ have distinguishable characteristics in their peel and/or pulp structures, which may be associated with moisture and weight loss during storage. Further studies should focus on determining the relationship between the structural characteristics of the peel and pulp of ‘Cheongsan’ and ‘Daebo’ and moisture and weight loss during storage.

Low-temperature storage is advantageous for preserving the nutrient quality of fruits; however, it can also trigger the accumulation of reactive oxygen species (ROS) such as superoxide, hydrogen peroxide, singlet oxygen, and hydroxyl radicals, which are involved in chilling injury [22,23]. Although cold stress induces oxidative damage in fruit, the ROS-scavenging system is activated to alleviate this damage through the help of antioxidative enzymes and secondary metabolites [23,24,25]. The ROS-scavenging system of various postharvested fruits is activated in response to low-temperature storage. Ascorbic acid is a major antioxidant that detoxifies ROS via the L-ascorbate glutathione cycle [26]. Although cold-treated tomato fruit maintained comparable levels of ascorbic acid, the genes related to ascorbic acid biosynthesis and redox reactions were expressed at higher levels in the tomato fruit stored at low temperatures than in the control [22]. Kiwifruit harvested at a stage less susceptible to chilling injury exhibited a higher abundance of antioxidant enzymes and lower levels of ROS [27]. Mume fruit stored at 1 °C had a higher antioxidant capacity and higher antioxidant enzyme activities, such as superoxide dismutase (SOD), catalase (CAT), and ascorbate peroxidase (APX), compared to those stored at 6 °C [28]. On the other hand, the SOD and CAT activities in the peel of mango fruits were weaker during storage at 4 °C than at 12 °C; meanwhile, the APX and guaiacol peroxidase activities did not significantly differ between the two storage temperatures [16]. It was also shown that the concentration of ascorbic acid in the peel of the mango fruits stored at 4 °C was lower than that in the fruits stored at 12 °C during the first 5 days of storage, although it recovered after 10 days [16]. Similarly, chilling injury in avocado fruit was more pronounced in those stored at 5 °C compared to 9 °C, with maximum antioxidant activity observed at 9 °C [29]. These previous studies showed that the underlying antioxidant mechanisms in response to cold stress differ among fruit cultivars and storage temperatures. Despite being smaller in size, the ‘Cheongsan’ fruits had higher total chlorophyll, carotenoid, flavonoid, and ascorbic acid per g of dry weight as well as higher DPPH- and ABTS-scavenging activities than the ‘Daebo’ fruits (Figure 6). However, the ‘Cheongsan’ fruits had lower DPPH- and ABTS-scavenging activities and ascorbic acid content in treatment L than at treatment ML after 2 weeks of storage. In terms of the decrease in the antioxidant activity and ascorbic acid content in the ‘Cheongsan’ fruits during the storage period, antioxidants such as ascorbic acid in the ‘Cheongsan’ fruits seem to be used to reduce cold-induced ROS at 3 °C, suggesting that ‘Cheongsan’ is markedly responsive to variations in low temperature than ‘Daebo’.

Hardy kiwifruit is known as a highly frost-resistant species that can tolerate temperatures as low as −30 °C in midwinter [30]. However, this characteristic should not be directly linked to the cold tolerance of hardy kiwifruit due to its small size and thin skin. Although fuzzy kiwi cultivars are more susceptible to chilling injury than hardy kiwi cultivars, ‘Hayward’ and ‘Haegeum’ can be cold stored for up to 90–150 days while maintaining fruit quality depending on the storage temperature and harvesting stage [31,32]. On the other hand, the storage period of hardy kiwifruit is shorter than that of fuzzy kiwifruit, with a storage duration of up to 30–60 days at 0–1 °C [6,33]. This suggests that hardy kiwifruit is more susceptible to cold storage than fuzzy kiwifruit. In this study, the low-temperature storage characteristics of two hardy kiwifruit cultivars, ‘Cheongsan’ and ‘Daebo,’ were determined. As ‘Cheongsan’ and ‘Daebo’ differ in size, their responses to low-temperature conditions varied in terms of changes in the physicochemical properties and antioxidant activity during the storage period. Specifically, although the ‘Cheongsan’ fruits contained higher levels of ascorbic acid and exhibited higher antioxidant activity than the ‘Daebo’ fruits, a relatively lower storage temperature (i.e., 3 °C rather than 5 °C) negatively affected the ascorbic acid content and antioxidant activity of the ‘Cheongsan’ fruits. Our findings suggest that changes in the antioxidants of hardy kiwifruit due to cold stress depends on the cultivar, with such cultivars potentially consuming their antioxidants to mitigate low-temperature damage. These results could be applied to establish suitable storage technologies to maintain the health-promoting compounds for each hardy kiwifruit cultivar.

## 4. Materials and Methods

### 4.1. Plant Material

The ‘Cheongsan’ and ‘Daebo’ fruits at the commercial maturity stage (7% brix, 100–105 and 115–120 days after full bloom, respectively) were harvested from a five-year-old tree (3 m × 3 m planting distance and pruned after harvesting at last year) after grafting from a hardy kiwifruit orchard in Wonju, Gangwon-do, Republic of Korea, and then immediately transported for 2 h to a low-temperature storage facility at the National Institute of Forest Science in Suwon, Gyeonggi-do, Republic of Korea. Uniform fruit samples without any physical damage were packed in perforated polyethylene terephthalate (PET) boxes (500–520 g per box, ‘Cheongsan’: approx. 35–40 fruits, ‘Daebo’: approx. 12–14 fruits) and placed in plastic containers (12 PET boxes per container). The containers—5 containers used in each hardy kiwifruit cultivar—were stored at 3 °C and 5 °C under approx. 85–90% relative humidity throughout the experiment.

### 4.2. Measurement of Quality Attributes

The weight loss was determined by weighing the fruit samples in the PET boxes throughout the storage period. The weight loss at different time points is expressed as a percentage of the initial weight. The firmness of the fruit samples was analyzed using a texture analyzer (CT3; AMETEK Brookfield, Middleboro, MA, USA) equipped with a 2 mm diameter flat probe at a compression speed of 1 mm/s. The probe was applied near the center of the flat surface of each fruit and penetrated to a depth of 10 mm. The results are expressed in Newtons (N). The TSSC (%) was measured with a digital refractometer (PR-101a; ATAGO, Tokyo, Japan). The TA was determined using a Titrator EasyPlus Easy pH (Mettler Toledo, Columbus, OH, USA), and the results are expressed as a percentage of anhydrous citric acid. The incidence of shrinking, browning, pitting, and decay is reported as a 6-point rate based on the percentage of symptoms on the surface of hardy kiwifruits: 0 = 0%, 1 = 1–20%, 2 = 21–40%, 3 = 41–60%, 4 = 61–80%, and 5 = 81–100% of the corresponding affected area on the fruit.

### 4.3. Color Measurement

The color characteristics of 20 randomly selected hardy kiwifruit samples were measured using a Minolta Chroma Meter (Model CR-400; Konica Minolta Optics, Osaka, Japan) at 3 equidistant points on the fruit samples. To describe the color characteristics of the fruit samples between treatments L and ML, the Hunter L* (lightness), a* (greenness < redness), and b* (blueness < yellowness) values were determined.

### 4.4. Sample Preparation for Determination of Health-Promoting Compounds

The hardy kiwifruit samples were freeze-dried using a freeze-dryer (MG-VFD20; MG industry, Gunpo, Republic of Korea) and then ground into a fine powder using an IKA Multidrive Basic (IKA Korea, Seoul, Republic of Korea). The powdered samples were used in the subsequent analyses.

### 4.5. Measurement of Starch and Soluble Sugar Content

The starch and soluble sugars were extracted using a method described previously [34]. In brief, the freeze-dried powdered sample (0.1 g) was homogenized with 2 mL 80% (*v*/*v*) ethanol. The homogenate was centrifuged at 10,000× *g* and 4 °C for 15 min. The supernatant was transferred to a new tube, and the resultant pellet was re-extracted twice with 80% (*v*/*v*) ethanol, as described above. The total supernatant was concentrated to 1 mL of the final volume using a vacuum centrifuge. The concentrated fraction was adjusted to 2 mL with distilled water, and an equal volume of chloroform was added to the concentrated fraction. The mixture was vigorously shaken to separate the aliphatic phase and centrifuged at 10,000× *g* at 4 °C for 15 min. The resultant upper water phase was used for sucrose, glucose, and fructose quantification. For starch extraction, 0.2 mL of 12 M HCl was added to the tube containing the dried pellet, following which 0.8 mL of dimethyl sulfoxide was added. After incubating at 60 °C for 30 min, 1 mL of water was added to the mixture, and the pH was adjusted to 4–5 with 5 N NaOH. The final volume of the mixture was adjusted to 4 mL and centrifuged at 10,000× *g* at 4 °C for 15 min. The resultant supernatant was used for starch quantification. The sugars and starch were quantified using enzymatic assay kits for sucrose/D-glucose/D-fructose and starch, respectively (R-Biopharm AG, Darmstadt, Germany).

### 4.6. Measurement of the Total Chlorophyll, Carotenoid, Flavonoid, and Phenolic Content

The freeze-dried powdered sample (0.2 g) was homogenized in 20 mL of 80% acetone and then filtered through Hyundai Micro filter paper No. 51 (Hyundai Micro, Seoul, Republic of Korea) to extract its chlorophyll, carotenoid, and flavonoid content. The total chlorophyll and carotenoid content of the filtered extract was measured using a spectrophotometer (Epoch2; Agilent Technologies, Santa Clara, CA, USA) at absorbance wavelengths of 662, 645, and 470 nm, which were used to calculate the total chlorophyll and carotenoid concentrations following the methods previously described by Arnon [35,36]. Meanwhile, the total flavonoid content of the filtered extract was measured using the method described by Zhishen et al. [37]. First, 1 mL of the filtered extract, 4 mL of distilled water, and 0.3 mL of 5% NaNO_2_ were mixed in a clean tube and then incubated at 25 °C for 5 min. Next, 0.3 mL of 10% AlCl_3_ was added to the tube; then, the tube was vortexed and incubated at 25 °C for 6 min. Afterwards, 2.4 mL of 1 M NaOH and 2.4 mL of distilled water were added to the tube. Lastly, the tube was vortexed, and the absorbance of the final solution was measured at 510 nm using a spectrophotometer (Epoch 2; Agilent Technologies, Santa Clara, CA, USA). The total flavonoid content was quantified from the absorbance values using a calibration curve constructed with catechin standards (Sigma-Aldrich, St. Louis, MO, USA). To extract the phenolic compounds, 0.2 g of the freeze-dried powdered sample was homogenized in 20 mL of MeOH:HCl (99:1, *v*/*v*). The mixture was centrifuged at 3000 rpm for 20 min at 10 °C. The total phenolic content of the supernatant was measured using the Folin–Ciocalteu reagent method [38]. In a clean tube, 0.1 mL of each of the supernatant, MeOH, and the Folin–Ciocalteu reagent were mixed together. The mixture was incubated for 6 min in the dark at 25 °C; after the brief incubation period, 0.7 mL of 20% Na_2_CO_3_ was added to the mixture. Subsequently, the resulting mixture was vortexed and incubated for 60 min in the dark at 25 °C. Finally, the mixture was centrifuged at 13,500 rpm for 3 min at 4 °C. The absorbance of the supernatant was measured at 735 nm using a spectrophotometer (Epoch 2; Agilent Technologies, Santa Clara, CA, USA). A gallic acid (Sigma-Aldrich, St. Louis, MO, USA) standard curve was used to calculate the total phenolic content.

### 4.7. Measurement of Antioxidant Activity

To extract antioxidants, 0.2 g of the freeze-dried powdered sample was homogenized in 20 mL of MeOH. The homogenized mixture was filtered through Hyundai Micro filter paper No. 51 (Hyundai Micro, Seoul, Republic of Korea), and the flow-through (final extract) was used to measure the antioxidant activity of the hardy kiwifruit samples. The DPPH-scavenging activity of the fruit samples was measured as described by Stoilova et al. [10] with some modifications. The final extract (0.2 mL) was added to 0.4 mM DPPH (1 mL). The extract (0.2 mL) with MeOH (1 mL) was used as the blank. The mixture was incubated at 25 °C for 30 min. After incubation, the absorbance of the mixture was measured at 517 nm using a spectrophotometer (Epoch 2; Agilent Technologies, Santa Clara, CA, USA), and the absorbance value was converted into the DPPH-scavenging activity (%). The ABTS-scavenging activity was determined according to the method described by Biglari, AlKarkhi, and Easa [11] with some modifications. ABTS (7 mL) was dissolved in potassium persulfate (2.45 mM) and incubated in the dark at 25 °C for 16 h. The ABTS solution was diluted in 70% ethanol to obtain an absorbance of approx. 1.5 at 734 nm. The final extract (40 μL) was added to the diluted ABTS solution (1 mL), and the reaction mixture was incubated at 25 °C for 6 min. Immediately after the reaction, the absorbance of the mixture was measured at 734 nm using a spectrophotometer (Epoch 2; Agilent Technologies, Santa Clara, CA, USA), and the absorbance value was converted into the ABTS-scavenging activity (%).

### 4.8. Determination of Ascorbic Acid Content

To extract ascorbic acid, 0.2 g of the freeze-dried powdered sample was homogenized in 10 mL of 2.5% (*w*/*v*) metaphosphoric acid. The mixture was centrifuged at 3000 rpm for 10 min at 25 °C. The supernatant was filtered using Hyundai Micro filter paper No. 51 (Hyundai Micro, Seoul, Republic of Korea), and the flow-through was passed through a syringe filter (0.45-μm pore size) prior to the analysis. Each sample was subjected to ultra-high performance liquid chromatography (UHPLC) analysis using an Ultimate 3000 UHPLC system (Thermo Fisher Scientific, Waltham, MA, USA) equipped with a Luna^®^C18 (150 × 3.0 mm, 3.0 μm) column (Phenomenex, Torrance, CA, USA). The UHPLC conditions were as follows: column temperature at 35 °C and UV detection at 254 nm. The isocratic mobile phase consisted of 20 mM KH_2_PO_4_ (pH 2.8), with a run time of 15 min at a flow rate of 0.2 mL/min. The amount of ascorbic acid in the extract was quantified using a calibration curve constructed with an authentic ascorbic acid standard (Sigma Aldrich, St. Louis, MO, USA).

### 4.9. Statistical Analysis

All the data are presented as the mean ± standard deviation. Student’s *t*-test was performed in GraphPad Prism 9 to determine whether the differences between the treatment groups were statistically significant.

## Data Availability

The data that support the findings of this study are included in this published article.
